# General practice and cardiac arrest community first response in Ireland

**DOI:** 10.1016/j.resplu.2021.100127

**Published:** 2021-05-05

**Authors:** Tomas Barry, Mary Headon, Martin Quinn, Mairead Egan, Siobhan Masterson, Conor Deasy, Gerard Bury

**Affiliations:** a School of Medicine, University College Dublin, Ireland; b Centre for Emergency Medical Science, University College Dublin, Ireland; c Health Service Executive, Ireland; d Clinical Strategy and Evaluation, National Ambulance Service, Health Service Executive, Ireland; e University College Cork, Ireland

**Keywords:** Emergency responders, Out-of-hospital cardiac arrest, Primary healthcare, General practice

## Abstract

**Background:**

In Ireland, the MERIT 3 scheme enables doctors to volunteer as cardiac arrest community first responders and receive text message alerts from emergency medical services (EMS) to facilitate early care.

**Aim:**

To establish the sustainability, systems and clinical outcomes of a novel, general practice based, cardiac arrest first response initiative over a four-year period.

**Methods:**

Data on alerts, responses, incidents and outcomes were gathered prospectively using EMS control data, incident data reported by responders and corroborative data from the national Out-of-Hospital Cardiac Arrest Registry.

**Results:**

Over the period 2016–2019, 196 doctors joined MERIT 3 and 163 (83.2%) were alerted on one or more occasions; 61.3% of those alerted responded to at least one alert. Volunteer doctors attended 300 patients of which 184 (61.3%) had suffered OHCA and had a resuscitation attempt. Responders arrived to OHCA before EMS on 75 occasions (40.8%), initiated chest compressions on seven occasions (3.8%), and brought the first defibrillator on 42 occasions (22.8%). Information on the first monitored rhythm was available for 149/184 (81.0%) patients and was shockable in 30/149 (20.1%); in 9/30 cases, shocks were administered by responders. The overall survival rate was 11.0% (national survival rate 7.3%). Doctors also provided advanced life support and were closely involved in decision making on ceasing resuscitation.

**Conclusion:**

The MERIT 3 initiative in Ireland has been sustained over a four-year period and has demonstrated the ability of volunteer doctors to provide early care for OHCA patients as well as more complex interventions including end-of-life care. Further development of this strategy is warranted.

## Introduction

Out-of-hospital cardiac arrest (OHCA) is the most time critical emergency whose outcomes depend on a chain of survival that integrates the community, emergency medical services and hospital care.^1^ In Europe, approximately 275,000 persons are treated for OHCA each year and OHCA represents a major cause of death.^2^, ^3^ Survival remains low with eight percent of patients discharged alive.^4^ In an effort to improve outcomes, increasing attention is being paid to the early links in the chain of survival, recognising that interventions that facilitate prompt cardiopulmonary resuscitation (CPR) and defibrillation in the community hold most promise.^5^, ^6^, ^7^ One such intervention is the mobilisation of community first responders. Community first responders (CFRs) are individuals who live or work within the community, are trained in basic life support and are mobilised to offer early OHCA care before the arrival of emergency medical services (EMS).^8^ The mobilisation of CFRs including firefighters, police officers, off duty medical staff and lay citizens has already been implemented in a number of regions.^9^, ^10^ Furthermore, this intervention has been shown to increase rates of early CPR and defibrillation and may improve survival.^8^, ^10^ Ireland's population of 4.7 million generates approximately 2500 OHCA resuscitation attempts each year with seven percent survival to hospital discharge.^11^, ^12^, ^13^, ^14^, ^15^ One third of Ireland's population live in rural areas (outside clusters of 1500 or more inhabitants) with longer EMS response times (9% statutory EMS call response interval <8 min or less compared to 33% in urban areas) and poorer outcomes following OHCA (3% survival to hospital discharge in rural areas compared to 6% in urban areas).^16^ For this reason, Irish statutory EMS have supported the training and mobilisation of volunteer CFRs and over 500 individual or group CFR resources are operational throughout Ireland.^17^ OHCA CFRs are activated by EMS generated text message alert and generally respond using their own cars. CFRs have fixed geographical coverage areas and are alerted to incidents (recognised as OHCA by EMS dispatch) that occur in these areas.

In Ireland, the role of healthcare professionals as volunteer OHCA responders has also been examined. Ireland has approximately 4000 general practitioners (GPs) who provide comprehensive primary care to the population across the lifespan.^18^ Irish GPs have a longstanding involvement in OHCA resuscitation and research. A previous study by our group from the MERIT 1 & 2 (Medical Emergency Responders Integration and Training) projects demonstrated that half of a cohort of 521 Irish general practices were involved in one or more cardiac arrest resuscitation attempts over a ten year period.^19^ Overall survival was 17% and most survivors experienced good functional outcome.^19^ Furthermore, resuscitation at a GP premises was associated with 47% survival to hospital discharge.^19^ In MERIT 1 & 2 GPs were supported to manage OHCA encountered in their day to day practice and there were no formal mechanisms linking GPs with statutory EMS dispatch. From 2015, a GP CFR initiative has allowed GPs to volunteer to receive EMS generated text message alerts to OHCA in their own communities via a structured national project called ‘MERIT 3′ (Medical Emergency Responders Integration and Training 3).

Our group previously reported early data describing the feasibility and initial uptake of the MERIT 3 initiative.^20^ We now report comprehensive experience of the MERIT 3 project over the period 2016–2019 inclusive, with comparable MERIT 1 & 2 data also described.

## Methods

GPs and a small number of other doctors (with backgrounds in Emergency Medicine) volunteer to participate in MERIT 3 as cardiac arrest CFRs. Responders can avail of regular immediate care training and are equipped with an AED and basic life support equipment.^21^ Volunteer responders specify a geo-location from which they will receive text message alerts to EMS recognised OHCA events occurring within a ten-kilometre radius. All responders who have specified a fixed point geolocation within a ten-kilometre radius of an OHCA event are automatically alerted by text message when OHCA is recognised by EMS dispatch. Responders can indicate specific hours during which they wish to receive OHCA alerts. Alerts include patient age, gender and the address of the incident including eircode (postcode). If in a position to attend, responders contact a dedicated EMS phone line to update EMS control and to obtain any additional information. Responders are under no obligation to attend. Responders mobilise to the scene using their personal car which has no special markings or emergency warning lights or sirens. Responders respect the standard rules of the road. MERIT 3 has received funding from the Department of Rural and Community Development and the medical charity Irish Community Rapid Response (ICRR). ‘ICRR subsidise the cost of ongoing immediate care training for responders including a bespoke one-day cardiac emergency care course. ICRR also fund automated external defibrillators (AEDs), basic life support and personal protective equipment (including basic airway management equipment, intravenous access equipment, gloves, eye protection and high visibility vest). All participating doctors must have an AED. Doctors do not receive any additional payments for participating in the project or responding to alerts. Participating GPs carry their own indemnity to provide medical care in the community and the care provided falls within responding doctor's routine scope of practice.

Data on alerts, responses, OHCA clinical care and outcomes were gathered prospectively. The National Emergency Operations Centre (NEOC) coordinates EMS response and manages emergency medical call taking and dispatch. NEOC notified the MERIT 3 research team of all alerts to participating responders on a quarterly basis. Individual responders provided reports on alerts received and anonymised clinical data on individual cases attended using a standardised incident report form based on the Utstein framework.^22^ Data supplied from NEOC on text alerts were validated by cross-referencing NEOC and Out-of-Hospital Cardiac Arrest Registry (OHCAR) data. OHCAR collates standardised information on the timings and outcomes of all OHCAs reported to it by all EMS in Ireland and therefore allows confirmation of whether a text alert resulted in a resuscitation attempt.^23^ This paper also reports on OHCAs managed within standard GP care by MERIT 1 & 2 GPs during the same period and compares this population with the novel MERIT 3 experience and with national data. The full details of the MERIT 1 & 2 parallel studies are reported elsewhere.^19^, ^24^, ^25^

Data were managed using Microsoft Excel and analysed for descriptive purposes. Proportions, medians, and ranges are reported as appropriate. Statistical analysis was conducted using IBM SPSS version 26. Ethical approval for the study was provided by the UCD Human Research Ethics Committee and both OHCAR and the National Ambulance Service have provided permission for access to data.

## Results

Table 1 demonstrates that from 2016 to 2019, MERIT 3 volunteer responder numbers rose from 107 to 190. Over the total study period 196 responders were active at any stage and 10 responders withdrew from the project. Four volunteer responders represented other medical specialities and the remainder were general practitioners. Over the study period MERIT 3 volunteer responders were alerted to 4163 individual incidents of presumed OHCA of which 1314 (31.6%) were ultimately recognised by OHCAR as OHCA with resuscitation attempts. Responders provided 361 individual responses resulting in 306 attendances involving 300 patients. In twenty-one instances a responder arrived on scene but did not see the patient as either sufficient EMS resources were already on scene or resuscitation efforts had already been terminated. In 34 cases, responders were ‘stood down’ by ambulance control before arriving at the scene. Of all responders potentially active at any point in the four year period 163/196 (83.2%) responders received one or more alerts (median 11, range 1–719, interquartile range 5–28) and 100/163 (61.3%) responders responded to one or more alerts (median 2, range 1–60, interquartile range 1–3).

**Table 1:** Responders available, alerts and responses 2016–2019.

Year	Active volunteer responders	Number of responders who received alert(s)	Number of responders who responded	Unique incidents	Responses to alerts	Patients attended
2016	107	75	27	818	65	51
2017	119	66	32	857	87	68
2018	182	131	54	1070	124	110
2019	190	136	42	1418	85	71

Total	196^a^	163^a^	100^a^	4163	361	300

a Active at any stage 2016–2019.

Table 2 summarises data on the 300 patients with whom responders had clinical contact. It demonstrates that responders arrived at scene before the EMS for 121/300 (40.3%) patients. An estimation of time to EMS arrival was provided by responders in sixty-nine cases and ranged from one to 60 min (median 10, interquartile range 4–20 min). An approximate patient age was available for 277/300 (92.3%) patients and ranged from the neonatal period to tenth decade (median 64, interquartile range 50–75 years old). Gender was available for 294/300 (98.0%) patients, 190 (64.6%) were male. In total 259/300 (86.3%) patients had suffered OHCA, of which 75/259 (29.0%) were found to be already deceased on the responder's arrival. The additional 41/300 (13.7%) patients had not suffered OHCA but presented with a wide range of clinical issues including alcohol intoxication (*n* = 6), syncopal episode (*n* = 5), unknown (*n* = 5), seizures (*n* = 3), trauma (*n* = 3) and drug overdose (*n* = 2). OHCA resuscitation was attempted for 184/300 (61%) patients.

**Table 2:** Summary data on 300 cases dealt with by responders.

Year	Patients attended	Arrival before EMS *n* (%)	OHCA^a^ *n* (% attended)	OHCA^a^ deceased on arrival *n* (% OHCA)	OHCA^a^ resuscitation attempted *n* (% attended)
2016	51	21 (41.2)	43 (84.3)	12 (27.9)	31 (60.8)
2017	68	23 (33.8)	61 (89.7)	17 (27.9)	44 (64.7)
2018	110	38 (34.5)	94 (85.5)	28 (29.8)	66 (60.0)
2019	71	39 (54.9)	61 (85.9)	18 (29.5)	43 (60.6)

Total	300	121 (40.3)	259 (86.3)	75 (29.0)	184 (61.3)

a Out-of-hospital cardiac arrest.

Of the 184 OHCA incidents where resuscitation was attempted the majority 120/184 (65.2%) were at a private residential location with an additional 14/184 (7.6%) at a nursing care facility. Overall, 32/184 (17.4%) incidents occurred in a public place, 11/184 (6%) at a sports facility and 5/184 (2.7%) at a workplace. One case (0.5%) occurred at a GP surgery and one further location (0.5%) was missing. Table 3 details key Utstein style variables considering the 184 cases in which resuscitation was attempted. Responders arrived in advance of EMS in 75/184 (40.8%) cases. Responders are known to have initiated CPR in 7/184 cases (3.8%) and brought the first AED in 42/184 cases (22.8%). Information on the first monitored rhythm was available for 149/184 (81.0%) patients and was shockable in 30/149 (20.1%); in 9/30 cases, shocks were administered by MERIT 3 responders. Ultimately 74/184 (40.2%) patients were known to be transported to hospital and 18/163 (11.0%) patients for whom survival outcome was available are known to have survived to hospital discharge. Survival outcome was not available for 21/184 patients.

**Table 3:** Resuscitation data on 184 out-of-hospital cardiac arrest (OHCA) cases.

Year	OHCA^a^ Resuscitation Attempts	Arrival before EMS^b^ n (%)	Brought 1st AED^c^ n (%)	Delivered 1st shock n (%)	Transported to Hospital n (%)	Survival Outcome Data Available n (%)	Survival to Hospital Discharge n (%)
2016	31	13 (41.9)	7 (22.6)	2 (6.5)	10 (32.3)	29 (93.5)	4 (13.8)
2017	44	15 (34.1)	7 (15.9)	2 (4.5)	20 (45.5)	38 (86.4)	5 (13.2)
2018	66	22 (33.3)	13 (19.7)	4 (6.1)	28 (42.4)	60 (90.9)	6 (10.0)
2019	43	25 (58.1)	15 (34.9)	1 (2.3)	16 (37.2)	36 (83.7)	3 (8.3)

Total	184	75 (40.8)	42 (22.8)	9 (4.9)	74 (40.2)	163 (88.6)	18 (11.0)

a Out-of-hospital cardiac arrest.

b Emergency medical services.

c Automated external defibrillator.

In addition to providing CPR and defibrillation, responders undertook various additional interventions while providing patient care in conjunction with EMS personnel. Over the study period responders performed bag-valve-mask (BVM) ventilation for 51/184 (27.7%) patients and face mask ventilation for 24/184 (13.0%) patients. An oropharyngeal airway was inserted by responders for 17/184 (9.2%) patients and a supraglottic airway for 42/184 (22.8%) patients. Responders acquired intravenous access for 43/184 (23.4%) patients and intra-osseous access for 18/184 (9.8%) patients. Adrenaline was the most commonly administered medication by responders for 48/184 (26.1%) patients followed by intravenous fluids for 20/184 (10.9%) patients. Amiodarone was administered by responders for 13/184 (7.1%) patients. Responders reported involvement in the decision to cease resuscitation for 80.0% (88/110) of cases where resuscitation was ceased on scene.

Table 4 summarises OHCA resuscitation attempts and outcomes over the period 2016–2019 from three sources – the MERIT 3 cohort reported here, the parallel MERIT 1 & 2 network of general practices who care for OHCA within their day-to-day practice (but who are not alerted to local OHCAs) and national data from OHCAR.^12^, ^13^, ^14^, ^15^ Within the MERIT 3 cohort 18/163 (11.0%) patients survived to hospital discharge, within the MERIT 1 & 2 cohort 33/128 (25.8%) survived to hospital discharge and at national level 703/9693 (7.3%) survived to hospital discharge.

**Table 4:** Summary of out-of-hospital cardiac arrest (OHCA) resuscitation attempts and outcomes 2016–2019.

	OHCA resuscitation attempts reported by GP CFRs					OHCA resuscitation attempts reported by routine general practice cohort (MERIT 1/2)					OHCAR national data			
Year	Active GP CF Rs	Cardiac arrest resus citation attempts	Survival outcome available	Survival to hospital discharge	Survival to hospital discharge %	Participating GP practices	Cardiac arrest resus citation attempts	Survival data available	Survived to hospital discharge	Survived to hospital discharge %	Total cardiac arrest resus citation attempts	Survival outcome available	Survival to hospital discharge	Total survival to hospital discharge %

2016	107	31	29	4	13.8	504	33	32	8	25.0	2389	2382	185	7.8
2017	119	44	38	5	13.2	509	41	40	7	17.5	2333	2325	152	6.5
2018	182	66	60	6	10.0	538	35	34	10	29.4	2442	2434	176	7.2
2019	190	43	36	3	8.3	538	22	22	8	36.4	2564	2552	190	7.4

Total	196^a^	184	163	18	11.0	538	131	128	33	25.8	9728	9693	703	7.3

a Active at any time.

Table 5 examines OHCAR documented survival rates among OHCA patients for whom a MERIT 3 alert was issued, comparing those who were attended by a MERIT 3 doctor and those who were not. Five cases recognised by the OHCAR dataset were excluded from this analysis as survival outcome was missing. In this comparison, survival was 11.1% (16/144) in those patients attended by a MERIT 3 responder and 7.4% (86/1165) in patients who were alerted to MERIT 3 responders but not attended (Odds Ratio 1.57, 95% Confidence Interval 0.89–2.76).

**Table 5:** Out-of-hospital cardiac arrest survival outcomes (OHCAR documented) in patients alerted to MERIT 3 responders.

	Survived to hospital discharge *n* (%)	Did not survive to hospital discharge *n* (%)	Total	Odds ratio survival (95% CI)
Attended by Merit 3 responder	16 (11.1)	128 (88.9)	144	1.57 (0.89–2.76)
Not attended by Merit 3 responder	86 (7.4)	1079 (92.6)	1165	

Total	102 (7.8)	1207 (92.2)	1309	

## Discussion

This study reports significant systems level achievements for the overall MERIT 3 initiative and demonstrates the impact of volunteer medical responders in individual cases of OHCA. Over the four-year period from 2016 to 2019, almost all MERIT 3 volunteers maintained their participation in the scheme, 83.2% were alerted and 61.3% of those alerted responded to one or more alerts. Participating volunteers received alerts at varying frequencies that are likely to reflect their differing geographical distributions. These data indicate a sustainable framework, within which doctors found it possible to respond to calls when they were available and make a wide range of interventions. Only ten of 196 volunteers left the scheme during the four-year period.

Volunteer responders attended 300 patients including 184 OHCAs where resuscitation was attempted, with an 11.0% survival rate. Responders arrived before EMS on 75 occasions (40.8%), initiated CPR on seven occasions (3.8%), brought the first AED on 42 occasions (22.8%) and delivered a first shock for 9 patients representing a significant proportion of those with a shockable rhythm. These findings demonstrate that MERIT 3 CFRs can deliver early critically important care before the arrival of EMS. Working with EMS advanced life support interventions were also provided on several occasions and doctors contributed to the majority of decisions to terminate resuscitation on scene. CPR was initiated infrequently however this likely reflects high levels of bystander/telephone CPR before responder arrival.

During the four-year period of this study, GPs working in distinct roles within MERIT 1 & 2 and MERIT 3 were involved in 315 cardiac arrests with resuscitation attempts, with 51 known survivors representing 7.3% of all survivors of OHCA in Ireland during the period. This reflects a key contribution by general practice to this significant health issue and underscores a continuing need to resource and prepare general practice for OHCA management.^26^

Positive contributions from CFR schemes have been seen in other health systems. Randomised trials from the Netherlands and Sweden found that OHCA CFRs increased CPR and defibrillation before EMS arrival.^8^, ^27^, ^28^ A further observational study from the Netherlands found that when text message alerted CFRs were activated in 893 OHCAs, a responder connected an AED in 184 cases and delivered a first shock in 76 cases.^29^ A study from Denmark found that smartphone app dispatched CFRs attended 438 OHCAs and arrived before EMS in 42% of cases, applied an AED in 20.8% and performed defibrillation in 4.3% of cases.^30^ Recently a ‘verified responder’ CFR program has been described in the United States, where off-duty EMS staff volunteer for the CFR role.^31^ We are not aware of any other CFR program that specifically targets doctors. Our data does however affirm previous work that suggested the addition of physician responders could widen population access to early OHCA care.^17^

When the overall CFR process including alerting, response and on scene care is considered, it is notable that a quarter of all patients attended during our study were already deceased on responders’ arrival and a further 14% had not suffered OHCA. Overall, almost 70% of OHCA alerts by EMS, did not have any attempt at OHCA resuscitation. These findings have significant implications for the organisation of EMS services and CFR initiatives as they suggest that attrition of resuscitation opportunity may occur at each of the various stages in this complex process. In Stockholm, Sweden when 4209 lay responders were alerted by smartphone app to 685 incidents of suspected OHCA, only 224 (32.7%) incidents ultimately represented OHCA resuscitation.^32^ A similar smartphone CFR initiative in Denmark found that only 508/819 (62.0%) EMS suspected OHCAs represented confirmed OHCA of which 45 cases represented end-of-life situations where resuscitation was not appropriate.^30^ Surveyed users of an OHCA smartphone CFR application in North America reported that 23% responded when alerted and of those 28% did not arrive on scene, and of those who did arrive on scene only 32% found a patient in OHCA.^33^ In France's Greater Paris area, over 40% of CFR smart phone app activations were noted to be inappropriate as either the patient was not in OHCA or was already deceased.^34^ Further efforts to tailor systems and allow community and EMS resources to be more closely matched with true resuscitation opportunities are warranted. It is possible that the disparities seen between alert and response numbers as well as resuscitation opportunities in our study reflect some inefficiencies in the current alerting process where CFRs are not geolocated in real time and are alerted automatically without consideration of other resources available or their proximity to the event. Modern smartphone applications integrate geolocation functionality and are increasingly being used to streamline this process and should be explored in the Irish context.^35^ Previous research has demonstrated that time to responder arrival can be reduced by a smart app based system.^36^ A further potential advantage of smart app technology is that it can allow more in-depth and accurate data capture regarding responses including accurate response time intervals.

In addition to preparing CFRs for the provision of basic life-support care, our results suggest that consideration must also be given to the reality of encountering and dealing with end-of-life situations. A key element of care provided by MERIT 3 responders related to decision making around end-of life care. Parallel qualitative research by our group has explored this issue in detail and highlighted the value of holistic, compassionate end-of-life care for patients and their families.^37^ Although decision making around cessation of resuscitation principally applies to medical responders, evidence suggests that the provision of supportive care around the circumstance of death is also a focus of lay responders.^37^, ^38^ Given the frequency at which CFRs are alerted to patients who are either already deceased or for whom resuscitation efforts will be terminated or are inappropriate, robust preparation and supports for this element of the role must be developed. Previous research has suggested that although the proportion of CFRs experiencing significant psychological ill effects related to participation is small, significant support is needed by some.^30^, ^37^, ^38^, ^39^, ^40^

## Limitations

In this study 41 patients were attended who presented with clinical issues other than OHCA. Our data collecting systems did not capture details of the care provided in these situations which may have been significant. Not all cases of OHCA where resuscitation was attempted by responders were captured in the OHCAR data set and survival outcome could not be obtained for all patients who were attended. A small number of survivors were notified to the research team via the GP responders only. Previously, in the region of ten percent of cases included in the OHCAR data set have been identified via missing case searches, however this proportion is expected to fall with the full implementation of an EMS electronic patient record system in Ireland. Although OHCA survival outcomes documented in this study demonstrate higher rates of survival than are achieved nationally, we cannot rule out a degree of response bias where responders may have been more likely to respond to situations with a greater chance of patient survival from the outset. Although less than one third of all alerts appeared to involve OHCA resuscitation opportunities, over half of cases where responders attended scene involved OHCA resuscitation. This does suggest some degree of response selectivity, the mechanisms of which are not yet clear but do require further exploration. While we have undertaken some comparisons of survival outcome considering patients attended or not attended by MERIT 3 responders, such comparisons have not adjusted for other factors known to influence survival outcomes.

## Conclusion

This general practice based, community first response initiative in Ireland facilitated early life support for OHCA patients both before the arrival of and in conjunction with EMS staff. Volunteer participation was sustained over a four-year period and clinical outcomes of OHCA care are comparable to those at national level. MERIT 3 could act as a template for a broader national system of volunteer professional early care of OHCA, supported by the health service.

## Competing interests

TB & GB are general practitioners with roles in cardiac arrest education, research, and clinical care. They declare no other competing interests.
